# Comparison of imaging findings of 714 symptomatic and asymptomatic temporomandibular joints: a retrospective study

**DOI:** 10.1186/s12903-023-02783-9

**Published:** 2023-02-07

**Authors:** Chuanjie Li, Qingbin Zhang

**Affiliations:** Guangzhou Key Laboratory of Basic and Applied Research of Oral Regenerative Medicine, Department of Temporomandibular Joint Surgery, Guangdong Engineering Research Center of Oral Restoration and Reconstruction, Affiliated Stomatology Hospital of Guangzhou Medical University, Guangzhou, 510000 China

**Keywords:** TMD, MRI, CBCT, Disc position, Condylar position, Disc morphology, Condylar position

## Abstract

**Background:**

The correlation between temporomandibular disorders (TMD) and imaging features remains unclear. This study compared the cone beam computed tomography (CBCT) and magnetic resonance imaging (MRI) features in the temporomandibular joints (TMJs) with and without TMD symptoms.

**Methods:**

The participants were recruited from the TMJ Diagnosis and Treatment Center from March 2022 to September 2022. Condylar morphology and condylar position were evaluated by CBCT. Disc morphology, disc position, and joint effusion were evaluated by T2-weighted image of MRI. The Chi-Square test and binary logistic regression analysis were carried out.

**Results:**

Eighty-two patients with bilateral symptoms, 196 patients with unilateral symptoms, and 79 asymptomatic participants received MRI and CBCT examination. There were significant differences in the distribution of sex, age, condylar morphology, condylar position, disc morphology, disc position, and joint effusion in symptomatic and asymptomatic TMJs (P < 0.05), which showed a positive correlation with symptoms (P < 0.05). In multiple logistic regression, 19–30-year-age group, > 30-year-age group, abnormal condylar morphology, posterior condylar position, disc displacement with reduction (DDWR), and disc displacement without reduction (DDWoR) were found to be statistically significant (P < 0.05). The odds of having symptomatic TMJ were 1.952 higher in the 19–30-year-age group and 1.814 higher in the > 30-year-age group when compared to those aged ≤ 18-year-age group. The odds of having symptomatic TMJ were 2.360 higher in persons with abnormal condylar morphology when compared to those with normal condylar morphology. The odds of having symptomatic TMJ were 2.591 higher in persons with posterior condylar position when compared to those with the normal condylar position. The odds of having symptomatic TMJ were 2.764 higher in persons with DDWR and 4.189 higher in persons with DDWoR when compared to those with normal disc position. The area under the curve of the model was 0.736 ± 0.019 (95% CI 0.700–0.773, P = 0.000), which indicated that the model has a good fitting effect.

**Conclusions:**

The imaging findings of TMJs were significantly different between symptomatic and asymptomatic TMJs. TMD is affected by multiple factors including > 19-year-age, abnormal condylar morphology, posterior condylar position, DDWR, and DDWoR, which could be risk factors for the development of TMD symptoms.

*Trial registration* This study was retrospectively registered on 28/03/2022 and endorsed by the Ethics Committee of Affiliated Stomatology Hospital of Guangzhou Medical University (LCYJ2022014).

## Background

Temporomandibular disorder (TMD) is a general term for a group of diseases involving temporomandibular joints (TMJs), masticatory muscles, and the surrounding tissues [1, 2]. TMD is the second most common musculoskeletal disorder that causes pain and disability [3, 4]. According to the Diagnostic Criteria for TMD (DC/TMD) Axis I, TMD could be divided into Group I: muscle disorders (including myofascial pain with and without mouth opening limitation); Group II: including disc displacement with or without reduction and mouth opening limitation; Group III: arthralgia, arthritis, and arthrosis [5]. TMD is often accompanied by the following symptoms: pain, decrease in the mouth opening, muscle or joint tenderness on palpation, limitation of mandibular movements, joint sounds, and otologic complaints like tinnitus, vertigo, or ear fullness, etc.

The treatment of atherogenic TMD usually includes non-surgical and surgical treatment. Non-surgical management options include non-steroidal anti-inflammatory drugs (NSAIDs), occlusal appliances, physical therapy, laser therapy, and some minimally invasive procedures such as intra-articular injections of hyaluronic acid (HA), corticosteroids, blood-derived products, and joint lavage through arthrocentesis [6–9]. Conservative treatment is now considered a first-choice therapy for TMD because of its low risk of side effects. In the case of severe acute pain or chronic pain resulting from serious disorders, inflammation, and/or degeneration pharmacotherapy, and minimally invasive/invasive procedures should be considered [10].

Internal derangement of the TMJ (TMJ ID) is considered the most common cause of TMD [11] and affects up to 80% of TMD patients. TMJ ID is attributed to an abnormal interaction of the articular disc, condyle, and joint eminence and may include a deformation, perforation, or displacement of the disc and/or posterior attachment of the disc [12]. Runci Anastasi et al. [13] believed that the disc-condyle complex is of great significance in TMD. Chantaracherd et al. [14] concluded that many people view IDs as a group of disorders that starts as disc displacement with reduction (DDWR), develops to disc displacement with reduction (DDWoR), and then to degenerative joint disease (DJD). Thus, the diagnosis of TMD requires not only sufficient clinical examination but also radiographic imaging examination.

Different radiographic imaging techniques have been proposed for evaluating the TMJ, such as computed tomography cone beam (CBCT), magnetic resonance imaging (MRI), TMJ arthrography, and panoramic radiography. TMJ intra-articular status was usually assessed by MRI and CBCT, although they have their shortcomings in enabling the clinician to effectively visualize the TMJ structures. At present, many studies are based on MRI or CBCT imaging data to evaluate its correlation with clinical symptoms [15–18]. However, these conclusions are not comprehensive enough.

MRI is the gold standard imaging method for evaluating the abnormalities of soft tissue in the TMJ, and masticatory muscles, and for determining the disc-condyle relationship [19]. MRI is usually used to evaluate the disc shape and disc position, joint effusion, the condylar bone marrow signal, and pannus formation [20]. MRI can also detect degenerative joint disease and condylar bone abnormalities, but could not efficiently detect the severity of the abnormalities [21]. There is currently a lack of large sample-size studies on the relationship between MRI and TMD clinical symptoms.

An important advantage of CBCT imaging of the TMJ is that it allows accurate measurements of the volume and the surface osseous changes, such as erosion, flatting, osteophytes, hypoplasia, sclerosis, and subchondral cyst with higher sensitivity, especially the early changes [22]. Additionally, it can be used to accurately assess the relative position of the condyle within the fossa. However, the morphology and position of the articular disc cannot be evaluated by CBCT. This means that CBCT alone cannot be used to evaluate the disc-condyle complex, but it is an important method to assist in the diagnosis of TMD. Based on the above-mentioned facts, this study aimed to compare the CBCT and MRI imaging features in the TMJs with and without TMD symptoms and to provide a reference for clinical diagnosis and treatment.

## Methods

### Ethical approval and study population

This study was endorsed by the Ethics committee of the Affiliated Stomatology Hospital of Guangzhou Medical University (LCYJ2022014). Informed consent to participate was obtained from all the participants and legally authorized representatives of minors age patients (< 16 Yrs). The selected participants were recruited from the patients referred for TMJ examination at the TMJ Diagnosis and Treatment Center of the Affiliated Stomatology Hospital of Guangzhou Medical University from March 2022 to September 2022. The CBCT was done for various reasons: evaluation of TMJ, impacted third molars, impacted teeth, implant site, pre-orthodontic assessment, or other reasons required CBCT. The MRI was done for the evaluation of the TMJ region. In the participants included in this retrospective study, both CBCT and MRI were taken before treatment and the time interval between the two scans was < 1 month.

### Inclusion and exclusion criteria

All participants received clinical examination according to Diagnostic Criteria for TMDs (DC/TMD) [5]. MRI and CBCT imaging data were obtained before treatment. Inclusion criteria were as follows: (1) both MRI and CBCT imaging performed within < 1-month intervals before TMD treatment; (2) sufficient valid clinical data exist for statistical analysis; (3) agree to participate in the study. Exclusion criteria were as follows: (1) tumor or fracture in TMJ area; (2) maxillofacial trauma, TMJ surgery, orthodontics treatment, rheumatism, or rheumatoid disease history; (3) severe morphological abnormalities of the condyle; (4) simply masticatory muscle problems; (5) restricted mouth opening due to interstitial infection and mucosal fibrosis; (6) severe psychological disorders; (7) poor quality scanning images; (8) contraindications to MRI or CBCT images; (9) disagree to participate in the study.

### Clinical examination

All participants were subjected to a standard clinical examination according to DC/TMD Axis I. Joint click, pain, and limited mouth opening were recorded. TMJ with at least one symptom is considered to be a symptomatic joint.

#### Joint sounds

Joint sounds were recorded by palpation on the TMJ region. Patients were asked to open their mouths as wide as possible and to do lateral, backward, and forward motions slowly 3 times respectively. Clicking, popping, or crepitation was recorded.

#### Pain

Pain intensity on a visual analog scale (VAS) was recorded. The VAS ranges from 0 to 10, where 0 indicates no pain and 10 indicates the worst pain.

#### Maximal interincisal opening (MIO)

The distance between the upper and lower incisors was measured when the patient opens his mouth. The limited mouth opening was defined as MIO < 42 mm.

### CBCT imaging

CBCT images were used to evaluate the condylar morphology and condylar position. Patients wear protective clothing when filming CBCT (Carestream CS9300, USA). Image reconstruction conditions were as follows: (1) the vertical fault was 60 mm in width and 0.3 mm in thickness; (2) the thickness of the deformed and coronal layer is 0.5 mm. The TMJ region in the oblique sagittal position was evaluated.

#### Condylar morphology

Condyles were divided into normal and abnormal in this study. Normal condyles have continuous cortical bone, while abnormal condyles are mainly divided into surface erosion, osteophyte, subcortical cyst, and generalized sclerosis [23]. The different condyle morphology was scored: 0 points represented normal condyle; 1 point represented the four abnormal types respectively; the cumulative value of abnormal types represented the degree of bone abnormality.

#### Condylar position

**Fig. 1:**
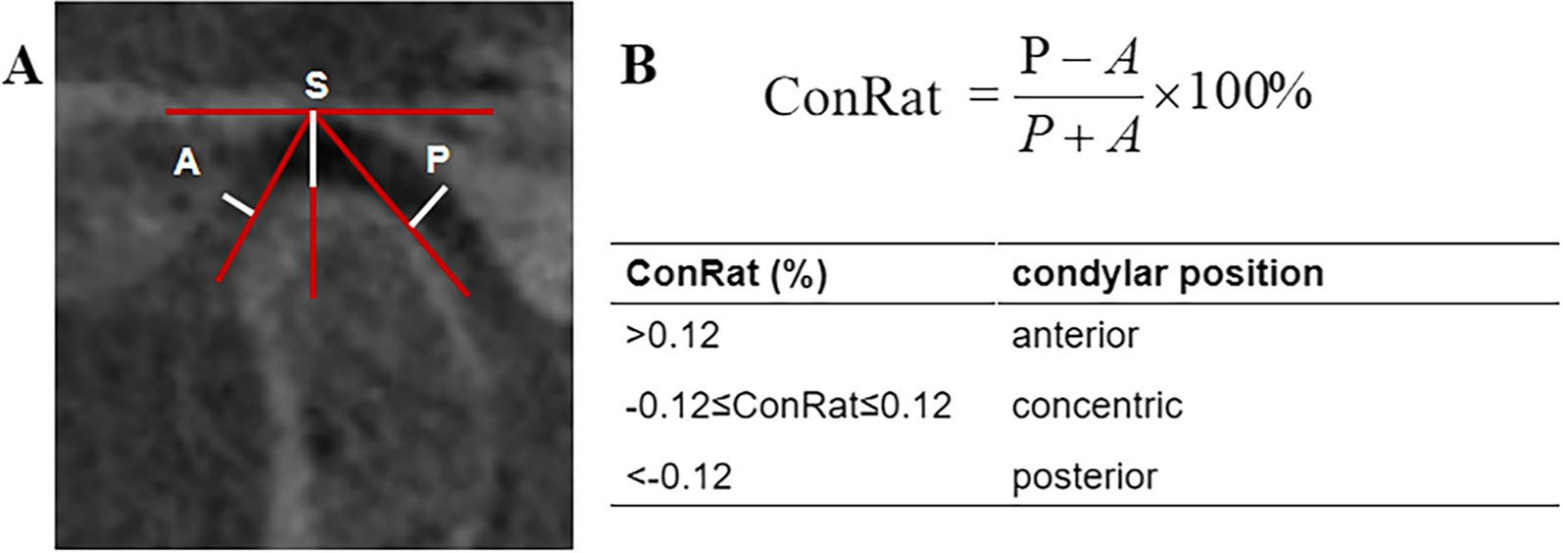
Condylar position. **A** Measurement of condyle position in CBCT imaging; **B** Calculation formula and interpretation method. A: anterior joint space; S: superior joint space; P: posterior joint space

Anterior joint space (AJS), superior joint space (SJS), and posterior joint space (PJS) in the oblique sagittal position were recorded by the same doctor as shown in Fig. 1A. The average of the three measurements was used for comparison. The condylar position within the glenoid fossa was classified according to the value of the condylar ratio, which was assessed on the basis of the formula presented by Pullinger and Hollende [24]. Condylar ratio = (P−A)/(P + A) × 100% (Fig. 1B). The explanation is as follows: (1) − 12% ≤ ConRat ≤ 12% indicates concentric position; (2) ConRat > 12% indicates anterior position; (3) ConRat < -12% indicates posterior position.

### MRI

MRI of bilateral TMJs with closed and open mouth position was obtained by a 1.5 T MRI scanner (UNITED IMAGING uMR, China). MRI images based on oblique sagittal adiposity-suppressed T2W1 were used to evaluate the disc morphology, disc position, and joint effusion.

#### Disc morphology

**Fig. 2:**
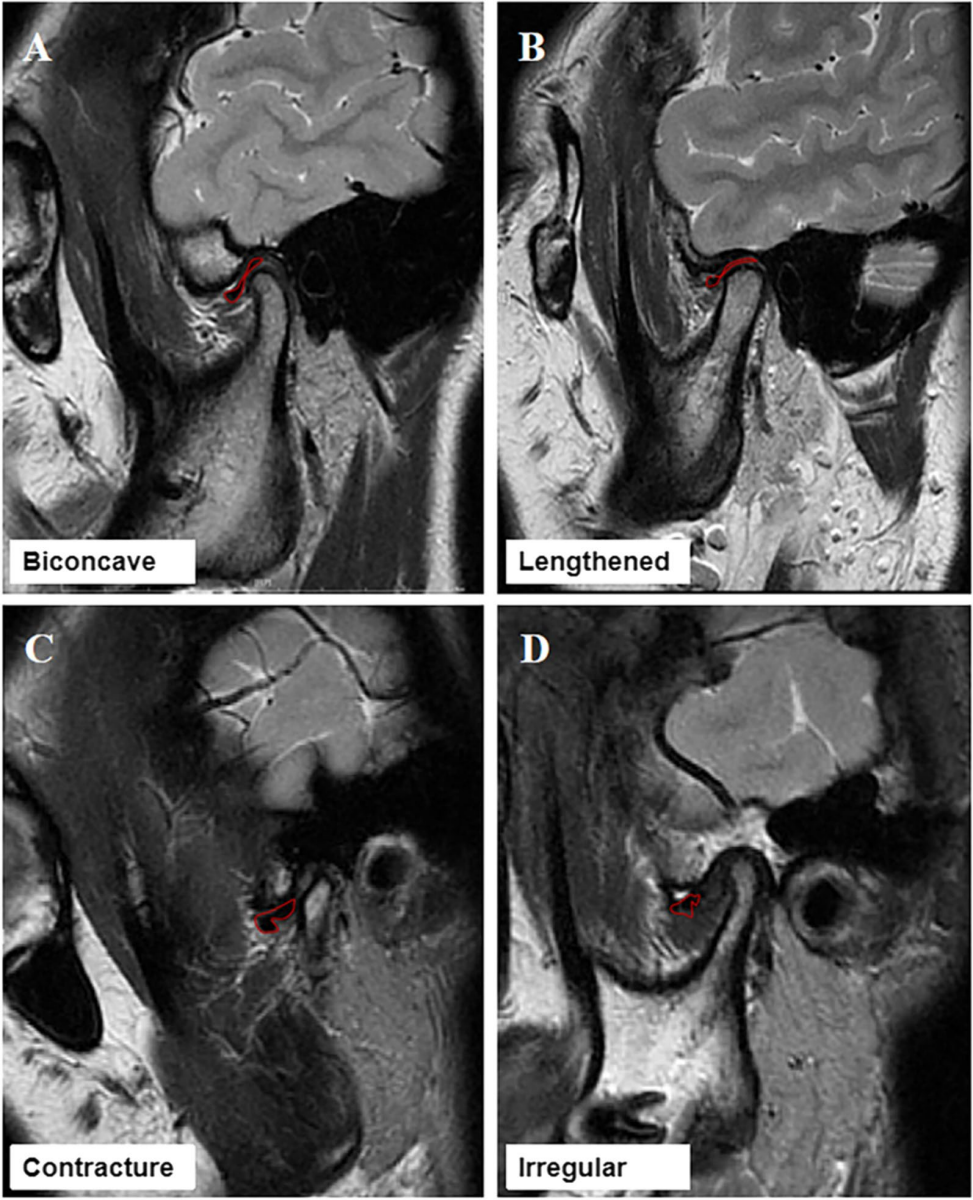
Disc morphology. **A** Biconcave, **B** lengthened, **C** Contracture, and **D** Irregular

The normal disc is biconcave, which has narrowed intermediate zone and fully visible posterior and anterior bands. In this study, the disc morphology was divided into the following four types according to the degree of folding as shown in Fig. 2: biconcave, lengthened, contracture, and irregular [25].

#### Disc position

**Fig. 3:**
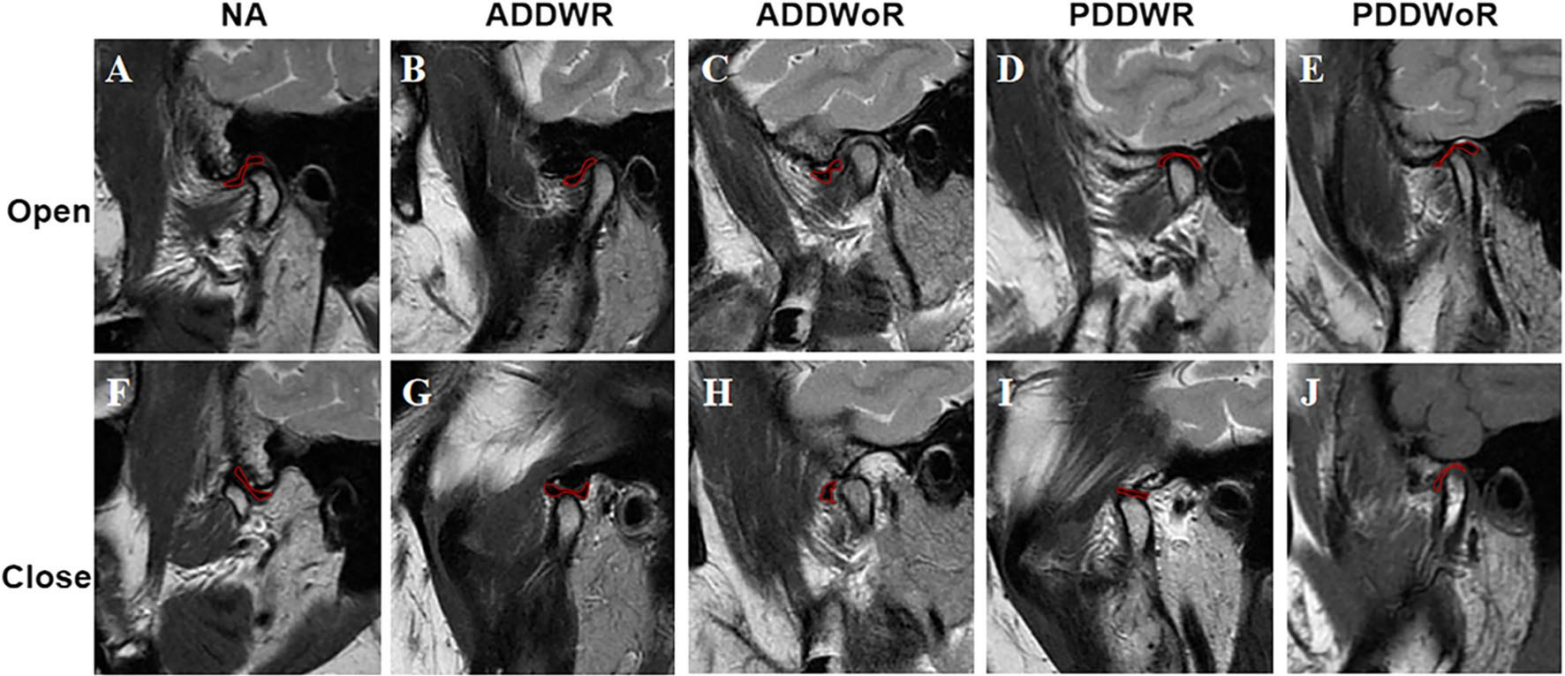
Disc position. **A–E** MRI images in the open-mouth position, **F–J** MRI images in the open-mouth position. Where: NA: normal; ADDWR: anterior disc displacement with reduction; ADDWoR: anterior disc displacement without reduction. PDDWR: posterior disc displacement with reduction; PDDWoR: posterior disc displacement without reduction

Disc position was evaluated at the closed-mouth position and the reduction of disc displacement was evaluated at the open-mouth position. The normal disc position is the posterior band located directly superior to the condylar head in the closed-mouth position [26]. Three types of disc position were evaluated: (1) − 15°–15° indicates the normal position; (2) > 15° indicates anterior disc displacement (ADD); (3) < -15° indicates posterior disc displacement (PDD) [27]. According to the reduction of disc position, disc position can be divided into the following five types (Fig. 3): (1) normal (NA); (2) anterior disc displacement with reduction (ADDWR); (3) anterior disc displacement without reduction (ADDWoR); (4) posterior disc displacement with reduction (PDDWR); (5) posterior disc displacement without reduction (PDDWoR)[28].

#### Joint effusion

**Fig. 4:**
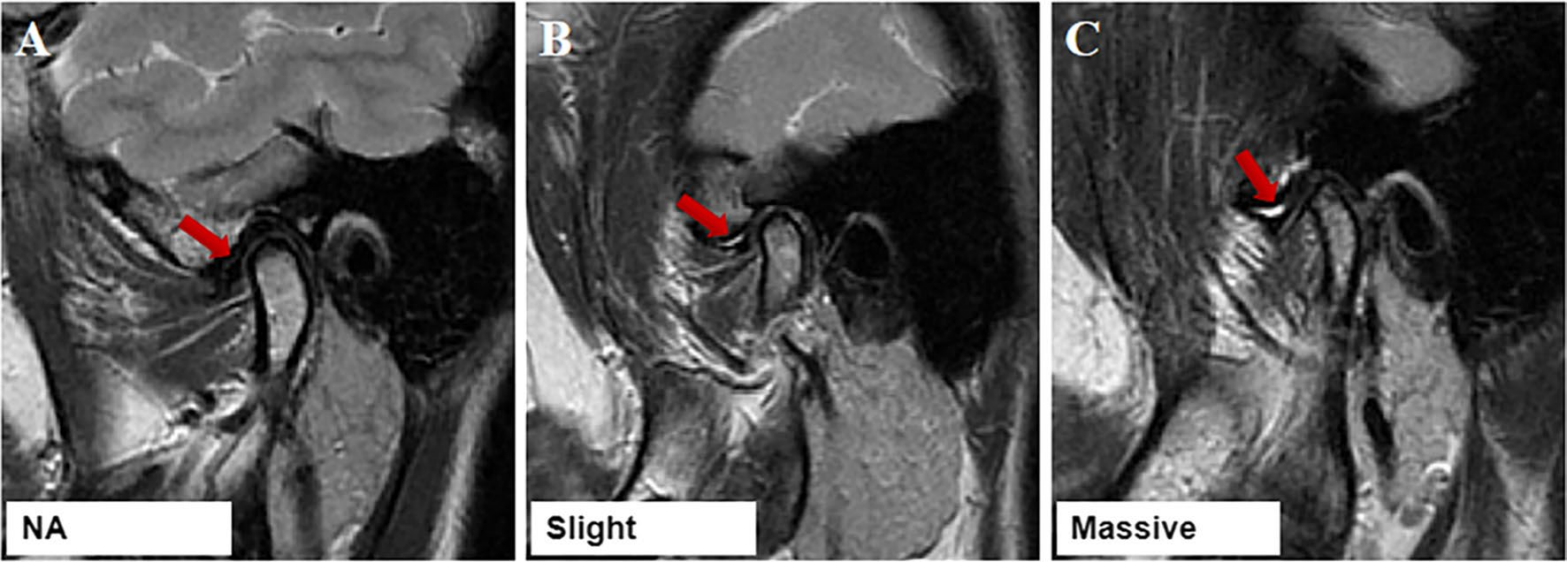
MRI images show joint effusion where the arrow indicates the effusion. **A** NA = no joint effusion; **B** slight joint effusion; **C** massive joint effusion. Where: NA: normal

There are three types of joint effusion included in this study (Fig. 4): (1) 0 point represented no joint effusion; (2) 1 point represented slight joint effusion: spot-like or linear high signal shadow; (3) 2 points represented massive effusion: high signal shadow of effusion pool is visible.

### Establishment of binary logistic regression model

The absence and presence of symptoms of TMJ were taken as dependent variables, and the Logistic regression values were 0 and 1, respectively. The following imaging features were used as independent variables to establish a Logistic model (including criteria P < 0.05 and excluding criteria P > 0.1). The forward method was used for stepwise regression. Criteria for assigning values: sex (male = 0 and female = 1); condylar morphology (normal = 0 and abnormal = 1); condylar position (anterior = 0, concentric = 0 and posterior = 1); disc morphology (normal = 0 and abnormal = 1); condylar position (biconcave = 0 and lengthened = 0, contracture = 0 and irregular = 1) and joint effusion (normal = 0, slight = 0, and massive = 1).

### Data analysis

Eligible data were analyzed by SPSS Statistics 23.0 software. The mean age and joint spaces were described by mean ± standard deviation. Independent-sample T test was performed for the comparison of mean age, AJS, SPS, and PJS between the symptomatic and asymptomatic TMJs. Continuous variables of three groups were analyzed by one-way ANOVA. Chi-Square test was performed for the distribution of sex, age, condylar morphology, condylar position, disc morphology, disc position, and joint effusion. Pearson Chi-Square test was performed for the expected frequency of less than 5. The correlations between the study variables were assessed using the Spearman correlation coefficient. Single-factor binary logistic regression and multiple logistic regression analysis were conducted between the asymptomatic and symptomatic TMJs. The receiver operating curve (ROC) was used to assess the predicting accuracy of this model. Furthermore, the area under the curve (AUC) was evaluated. P < 0.05 was considered statistically significant.

## Results

### Basic information

A total of 357 participants (286 females and 71 males) with 714 TMJs were included in the study and divided into three groups: symptoms on one side (54.9%, 196/357), symptoms on both sides (23.0%, 82/357), and no symptoms on both sides (22.1%, 79/357), with the mean age of 27.27 ± 11.50, 26.38 ± 11.44, and 22.94 ± 9.51 years, respectively (P = 0.014) (Table 1). There were significant differences in the distribution of sex and age in different symptom sides (χ^2^ = 13.105, P = 0.001 and χ^2^ = 11.583, P = 0.021). In the No symptoms on both sides group, the 19–30-year-age-group ranked first followed by ≤ 18 and > 30-year-age groups. In Symptoms on one side and Symptoms on both sides groups, the 19–30-year-age group ranked first followed by > 30 and ≤ 18-year-age groups. The mean age of patients with symptoms on one side was significantly higher than that of patients with no symptoms and symptoms on both sides (F = 4.338 and P = 0.014).

**Table 1:** Participant’s characteristics

	No symptoms on both sides (n = 79)	Symptoms on one side (n = 196)	Symptoms on both sides (n = 82)	P
Sex n (%)	52 (65.8)	164 (83.7)	70 (85.4)	χ^2^ = 13.105, P = 0.001
Age Y mean ± SD	22.94 ± 9.51	27.27 ± 11.50	26.38 ± 11.44	F = 4.338, P = 0.014
≤ 18 Y n (%)	28 (35.4)	39 (19.9)	13 (15.9)	χ^2^ = 11.583, P = 0.021
19–30 Y n (%)	37 (46.8)	108 (55.1)	52 (63.4)	
> 30 Y n (%)	14 (17.7)	49 (25.0)	17 (20.7)	

The ratio was obtained from Pearson Chi-Square test analysis and the mean age was obtained from one-way ANOVA. Where: Y = year. Significant difference was set at P < 0.05

Secondly, as shown in Table 2, all the 714 TMJs included in the study were divided into two groups according to the presence or absence of clinical symptoms: asymptomatic (n = 354, 25.34 ± 10.85 years) and symptomatic (n = 360, 26.88 ± 11.50 years) (P = 0.066). There were significant differences in AJS and SPS between asymptomatic and symptomatic groups, but no significant differences in PJS (P = 0.741). The value of AJS in the asymptomatic group was significantly lower than that in the symptomatic group (P = 0.000). The value of SJS in the asymptomatic group was significantly higher than that in the symptomatic group (P = 0.038).

**Table 2:** Comparison between the symptomatic and asymptomatic side of TMJ

	Asymptomatic (n = 354)	Symptomatic (n = 360)	t	CI (95%)		P
				Lower	Upper	
Age	25.34 ± 10.85	26.88 ± 11.50	− 1.838	− 3.182	0.105	0.066
AJS	2.53 ± 0.81	2.77 ± 0.87	− 3.867	− 0.367	− 0.120	0.000
SPS	2.96 ± 0.93	2.82 ± 0.87	2.080	0.008	0.273	0.038
PJS	2.35 ± 0.81	2.33 ± 0.88	0.331	− 0.103	0.144	0.741

Mean age and joint spaces were described by mean ± standard deviation. An Independent-samples T test was performed for the comparison of mean age, AJS, SPS, and PJS between the symptomatic and the asymptomatic side. CI: confidence interval; AJS: anterior joint space; SJS: superior joint space; PJS: posterior joint space. Significant difference was set at < 0.05

### Comparison of demographic/disease features with symptomatic and asymptomatic groups

The distribution and the indirect relation of demographic/disease features with the occurrence of symptoms were performed by Pearson Chi-Square test and Spearman correlation coefficient respectively, as shown in Table 3. There were significant differences in the distribution of sex (χ^2^ = 8.554, P = 0.000), age (χ^2^ = 8.084, P = 0.018), condylar morphology (χ^2^ = 64.693, P = 0.000), condylar position (χ^2^ = 14.681, P = 0.001), disc morphology (χ^2^ = 65.170, P = 0.000), disc position (χ^2^ = 83.642, P = 0.000) and joint effusion (χ^2^ = 53.911, P = 0.000) in symptomatic and asymptomatic TMJs, and showed positively correlated with symptoms (P < 0.05). The proportion of women in the symptomatic group was higher than that in the asymptomatic group, and sex was positively correlated with symptoms (r = 0.109, P = 0.003). In the asymptomatic group, the 19–30-year-age group ranked first followed by ≤ 18 and > 30-year-age groups. In the symptomatic group, the 19–30-year-age group ranked first followed by > 30 and ≤ 18-year-age groups. And age was positively correlated with symptoms (r = 0.075 and P = 0.044).

**Table 3:** Distribution of sex, age, and imaging features of symptomatic and asymptomatic sides

	Parameter	Asymptomatic (n = 354)	Symptomatic (n = 360)	P	r, P
Sex n (%)	Female	268 (75.7)	304 (84.4)	χ^2^ = 8.554, P = 0.003	r = 0.109, P = 0.003
Age n (%)	≤ 18 Y	95 (26.8)	65 (18.1)	χ^2^ = 8.084, P = 0.018	r = 0.075, P = 0.044
	19–30 Y	182 (51.4)	212 (58.9)		
	> 30 Y	77 (21.8)	83 (23.1)		
Condylar morphology n (%)	0	266 (75.1)	176 (48.9)	χ^2^ = 64.693, P = 0.000	r = 0.281, P = 0.000
	1	67 (18.9)	121 (33.6)		
	2	20 (5.6)	32 (8.9)		
	3	1 (0.3)	29 (8.1)		
	4	0 (0)	2 (0.6)		
Condylar position n (%)	Anterior	71 (20.1)	58 (16.1)	χ^2^ = 14.681, P = 0.001	r = 0.130, P = 0.001
	Concentric	173 (48.9)	140 (38.9)		
	Posterior	110 (31.1)	162 (45.0)		
Disc morphology n (%)	Biconcave	164 (46.3)	73 (20.3)	χ^2^ = 65.170, P = 0.000	r = 0.279, P = 0.000
	Lengthened	46 (13.0)	36 (10.0)		
	Contracture	95 (26.8)	169 (46.9)		
	Irregular	49 (13.8)	82 (22.8)		
Disc position n (%)	NA	148 (41.8)	47 (13.1)	χ^2^ = 83.642, P = 0.000	r = 0.300, P = 0.000
	ADDWR	74 (20.9)	84 (23.3)		
	ADDWoR	121 (34.2)	222 (61.7)		
	PDDWR	10 (2.8)	6 (1.7)		
	PDDWoR	1 (0.3)	1 (0.3)		
Joint effusion n (%)	0	331 (93.5)	264 (73.3)	χ^2^ = 53.911, P = 0.000	r = 0.273, P = 0.000
	1	23 (6.5)	86 (23.9)		
	2	0 (0.0)	10 (2.8)		

The data were obtained from the Pearson Chi-Square test and Spearman correlation coefficient analysis

Significant difference was set at P < 0.05

*Y* year; *NA* normal; *ADDWR* anterior disc displacement with reduction; *ADDWoR* anterior disc displacement without reduction; *PDDWR* posterior disc displacement with reduction; *PDDWoR* posterior disc displacement without reduction

Zero ranked first followed by 1, 2, 3, and 4 in both the asymptomatic and symptomatic group, and condylar morphology was positively correlated with symptoms (r = 0.281, P = 0.000). The proportion of normal condyle in the asymptomatic group was significantly higher than that in the symptomatic group. In the asymptomatic group, concentric ranked first followed by posterior and anterior. In the symptomatic group, the posterior ranked first followed by concentric and anterior. And the condylar position was positively correlated with symptoms (r = 0.130 and P = 0.001).

In the asymptomatic group, biconcave ranked first followed by contracture, irregular, and lengthened. In the symptomatic group, contracture ranked first followed by irregular, biconcave, and lengthened. And disc morphology was positively correlated with symptoms (r = 0.279 and P = 0.000). In the asymptomatic group, NA ranked first followed by ADDWoR, ADDWR, PDDWR, and PDDWoR. In the symptomatic group, ADDWoR ranked first followed by ADDWR, NA, PDDWR, and PDDWoR. And disc position was positively correlated with symptoms (r = 0.300 and P = 0.000). Zero ranked first followed by 1 and 2 in both the asymptomatic and symptomatic groups, and joint effusion was positively correlated with symptoms (r = 0.273, P = 0.000).

### Risk factors for TMD symptoms

Firstly, a single-factor binary logistic regression analysis was conducted between the asymptomatic and symptomatic groups. There was no significant difference in condylar positions, lengthened disc morphology, DDWR, or joint effusion between the two groups (P > 0.05). There were significant differences in sex, 19–30 years, > 30 years, condylar morphology, contracture disc morphology, irregular disc morphology, and DDWoR between the two groups (P < 0.05, Table 4).

**Table 4:** Results of binary logistic regression analysis

		Univariable			Multivariable		
		OR	CI (95%)	P	OR	CI (95%)	P
Sex	Female	1.742	1.198–2.533	0.004			0.747
Age	≤ 18 Y			0.018			0.005
	19–30 Y	1.702	1.173–2.471	0.005	1.952	1.296–2.939	0.001
	> 30 Y	1.575	1.012–2.452	0.044	1.814	1.113–2.954	0.017
Condylar morphology	Abnormal	0.262	0.190–0.361	0.000	2.360	1.613–3.453	0.000
Condylar position	concentric			0.303			0.000
	anterior			0.671	0.661	0.413–1.059	0.085
	posterior			0.172	1.712	1.192–2.461	0.004
Disc morphology	biconcave			0.001			0.225
	lengthened	0.963	0.580–1.601	0.885			0.414
	contracture	0.655	0.460–0.933	0.019			0.187
	irregular	0.429	0.277–0.664	0.000			0.672
Disc position	NA			0.000			0.000
	DDWR	0.876	0.580–1.325	0.531	2.764	1.737–4.397	0.000
	DDWoR	0.523	0.366–0.746	0.000	4.189	2.693–6.515	0.000
Joint effusion	0			0.454			
	1			0.302			
	2			0.507			

Binary logistic regression analysis was conducted between the asymptomatic and symptomatic groups

Significant difference was set at P < 0.05

*Y* year; *NA* normal; *DDWR* disc displacement with reduction; *DDWoR* disc displacement without reduction; *OR* odds ratio, *CI* confidence interval

Then, sex, age, condylar morphology, condylar position, disc morphology, and disc position were included in the multifactor analysis according to clinical knowledge. In multiple logistic regression, the six factors were found to be statistically significant: 19–30 years [P = 0.001, OR = 1.952, CI (95%) = (1.296–2.939)], > 30 years [P = 0.017, OR = 1.814, CI (95%) = (1.113–2.954)], abnormal condylar morphology [P = 0.000, OR = 2.360, CI (95%) = (1.613–3.453)], posterior condylar position [P = 0.000, OR = 2.591, CI (95%) = (1.578–4.253)], DDWR [P = 0.000, OR = 2.764, CI (95%) = (1.737–4.397)], and DDWoR [P = 0.000, OR = 4.189, CI (95%) = (2.693–6.515)].

Hence, the odds of having symptomatic TMJ were 1.952 higher in the 19–30-year-age group and 1.814 higher in the > 30-year-age group when compared to the ≤ 18-year-age group. The odds of having symptomatic TMJ were 2.360 higher in persons with abnormal condylar morphology when compared to those with normal condylar morphology. The odds of having symptomatic TMJ were 2.591 higher in persons with posterior condylar position when compared to those with the normal condylar position. The odds of having symptomatic TMJ were 2.764 higher in persons with DDWR and 4.189 higher in persons with DDWoR when compared to those with normal disc positions.

### ROC curve analysis of the model

**Fig. 5:**
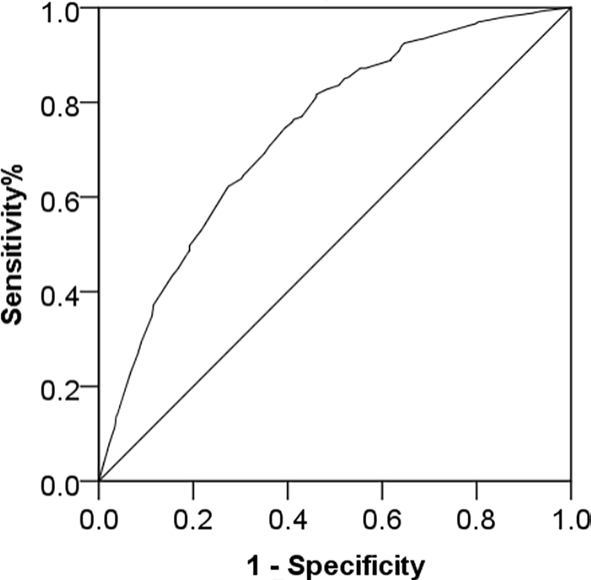
ROC curve analysis of the model

The ROC was used to assess the predicting accuracy of this model as shown in Fig. 5. The area under the curve (AUC) was evaluated. The AUCs of the model were 0.736 ± 0.019 (95% CI 0.700–0.773, P = 0.000), which indicated that the model has a good fitting effect and can be used to predict the occurrence of TMJ symptoms.

## Discussion

This study was conducted to compare the CBCT and MRI imaging features in the TMJs with and without TMD symptoms. A total of 357 participants with 714 TMJs were included in the study. There were significant differences in the distribution of sex, age, condylar morphology, condylar position, disc morphology, disc position, and joint effusion in symptomatic and asymptomatic TMJs, and these features were positively correlated with TMD symptoms. In multiple logistic regression, the 19–30-year-age group, > 30-year-age group, abnormal condylar morphology, posterior condylar position, DDWR, and DDWoR were found to be statistically significant.

Patients with TMD symptoms are diagnosed over a broad age range, but a peak occurs between 20 and 40 year-age [29]. There was no statistically significant difference in mean age between the asymptomatic and symptomatic groups, possibly because patients in the unilateral asymptomatic group overlapped in both groups. In this study, the odds of having symptomatic TMJ were 1.952 higher in the 19–30-year-age group and 1.814 higher in the > 30-year-age group when compared to the ≤ 18-year-age group.

Although there was a poor correlation between condylar changes (as observed on CBCT images), pain, and symptoms in TMJ OA [30]. Many studies have confirmed the correlation between condylar bone changes and articular disc displacement and believed that it is related to TMD symptoms [31, 32]. Consistent with previous research results, normal condylar morphology was 75.1% in the asymptomatic group and 48.9% in the symptomatic group in this study. The odds of having symptomatic TMJ were 2.360 higher in persons with abnormal condylar morphology when compared to those with normal condylar morphology.

The relationship between condylar location and TMD symptoms is controversial. Shokri et al. observed that the posterior condylar position is more common in TMD patients [33]. And Yasa et al. found that the presence of TMD is associated with the condylar position in the TMJ [34]. However, Ma et al. [35] compared the condylar position in symptomatic and asymptomatic participants with and without chewing side preference and found that the condylar position is not a strong indicator. Paknahad et al. found the condylar position is associated with different severity of TMD [36]. However, the sample sizes of both studies were small. In this study, there were significant differences in AJS and SPS between asymptomatic and symptomatic groups, but no significant differences in PJS. The posterior condyle was the most common in the symptomatic group, while the concentric condyle was the most common in the asymptomatic group. The odds of having symptomatic TMJ were 2.591 higher in persons with posterior condylar position when compared to those with the normal condylar position.

Disc changes can be seen in up to 80% of TMD patients, but 30% of asymptomatic cases have similar findings [37]. Articular disc morphology was divided into 4 types: biconcave, lengthened, contracture, and irregular. The biconcave disc was the most common in the asymptomatic group, while the contracture disc was the most common in the symptomatic group. But the disc morphology did not seem to be the risk factor for TMD symptoms. Disc displacement on MRI correlated well with the presence or absence of clinical signs and symptoms of TMD [18, 38, 39]. The study found that the disc position was positively correlated with symptoms. The odds of having symptomatic TMJ were 2.764 higher in persons with DDWR and 4.189 higher in persons with DDWoR when compared to persons with normal disc position.

Joint effusion is the state of accumulation of joint fluid in the joint spaces and surrounding tissue [40]. Our present study found that there was a significant difference in the distribution of joint effusion in different types of disc morphology [25]. In this study, the proportion of participants without joint effusion in asymptomatic and symptomatic groups was 93.5% and 73.3%, respectively. Consistent with the results of Khawaja’s study [41], joint effusion is not a risk factor for TMD symptoms.

## Conclusions

Unilateral symptoms of TMJs are more common in TMD. The imaging findings of TMJs were significantly different between symptomatic and asymptomatic TMJs. TMD is affected by multiple factors including > 19-year-age, abnormal condylar morphology, posterior condylar position, DDWR, and DDWoR, which could be risk factors for the development of TMD symptoms. Further studies are needed to confirm the end point of TMD treatment.

## Data Availability

The datasets generated and/or analyzed during the current study are not publicly available due to ethical concerns but are available from the corresponding author on reasonable request.

## Abbreviations

TMD: Temporomandibular disorder
TMJ: Temporomandibular joint
NA: Normal
ADD: Anterior disc displacement
PDD: Posterior disc displacement
ADDWR: Anterior disc displacement with reduction
ADDWoR: Anterior disc displacement without reduction
CBCT: Cone beam computed tomography
MRI: Magnetic resonance imaging
ROC: Receiver operating curve
AUC: Area under the curve
T2WI: T2-weighted image
VAS: Visual analog scale
MIO: Maximal interincisal opening
